# Selective inhibition of BCL-2 is a promising target in patients with high-risk myelodysplastic syndromes and adverse mutational profile

**DOI:** 10.18632/oncotarget.24775

**Published:** 2018-04-03

**Authors:** Veronika Reidel, Johanna Kauschinger, Richard T. Hauch, Catharina Müller-Thomas, Niroshan Nadarajah, Rainer Burgkart, Burkhard Schmidt, Dirk Hempel, Anne Jacob, Julia Slotta-Huspenina, Ulrike Höckendorf, Christian Peschel, Wolfgang Kern, Torsten Haferlach, Katharina S. Götze, Stefanie Jilg, Philipp J. Jost

**Affiliations:** ^1^ Medizinische Klinik für Hämatologie und Internistische Onkologie, Klinikum rechts der Isar, Technische Universität München, Munich, Germany; ^2^ Munich Leukemia Laboratory (MLL), Munich, Germany; ^3^ Klinik für Orthopädie und Sportorthopädie, Klinikum rechts der Isar, Technische Universität München, Munich, Germany; ^4^ Gemeinschaftspraxis Hämato-Onkologie Pasing, Munich, Germany; ^5^ Onkologisches Zentrum Donauwörth, Donauwörth, Germany; ^6^ Institut für Pathologie, Klinikum rechts der Isar, Technische Universität München, Munich, Germany; ^7^ Deutsche Konsortium für translationale Krebsforschung (DKTK) of the German Cancer Research Center (DKFZ), Heidelberg, Germany

**Keywords:** apoptosis, BCL-2 family, ABT-199, myelodysplastic syndromes, myeloid malignancy, Autophagy

## Abstract

Somatic mutations in genes such as *ASXL1*, *RUNX1*, *TP53* or *EZH2* adversely affect the outcome of patients with myelodysplastic syndromes (MDS). Since selective BCL-2 inhibition is a promising treatment strategy in hematologic malignancies, we tested the therapeutic impact of ABT-199 on MDS patient samples bearing an adverse mutational profile. By gene expression, we found that the level of pro-apoptotic BIM significantly decreased during MDS disease progression in line with an acquired resistance to cell death. Supporting the potential for ABT-199 treatment in MDS, high-risk MDS patient samples specifically underwent cell death in response to ABT-199 even when harbouring mutations in *ASXL1*, *RUNX1*, *TP53* or *EZH2*. ABT-199 effectively targeted the stem- and progenitor compartment in advanced MDS harbouring mutations in *ASXL1*, *RUNX1*, *TP53* or *EZH2* and even proved effective in patients harbouring more than one of the defined high-risk mutations. Moreover, we utilized the protein abundance of BCL-2 family members in primary patient samples using flow cytometry as a biomarker to predict ABT-199 treatment response. Our data demonstrate that ABT-199 effectively induces apoptosis in progenitors of high-risk MDS/sAML despite the presence of adverse genetic mutations supporting the notion that pro-apoptotic intervention will hold broad therapeutic potential in high-risk MDS patients with poor prognosis.

## INTRODUCTION

Somatic mutations of *ASXL1*, *RUNX1*, *TP53* or *EZH2* have a strong negative impact on the outcome of MDS patients [1]. Established therapeutic approaches such as hypomethylating agents [2–4] or allogeneic stem cell transplantation [5] only partially overcome the reduced survival rates and innovative therapeutic strategies are urgently needed.

Aberrant blockade of cell death represents a hallmark of myeloid neoplasia [6–10]. The anti-apoptotic protein BCL-2 provides protection against various pro-apoptotic stresses by blocking cell death induction upstream of mitochondrial permeabilization [11]. In higher-risk Myelodysplastic Syndromes (MDS), the induction of clinical remission critically depends on the capacity of the treatment regime to overcome the acquired apoptotic resistance. This is due to the finding that the balance between pro-survival and pro-apoptotic proteins of the BCL-2 family is substantially disturbed during disease progression [12, 13]. However, the exact mechanism controlling the deregulated cell death induction in MDS is not fully understood [14, 15].

We have recently shown that pharmacologic inhibition of BCL-2 induces cell death in MDS [7]. ABT-199 selectively drives higher-risk MDS stem/progenitor cells into apoptotic cell death, while sparing progenitors from low-/intermediate-risk patients as well as healthy control progenitors [7]. Hence, incorporation of ABT-199 into treatment strategies for higher-risk MDS patients likely represents an innovative treatment strategy, which is currently being tested in clinical trials. Now it is of special interest whether ABT-199 can overcome apoptotic resistance also in high-risk patients with documented mutations of *ASXL1*, *RUNX1*, *TP53* or *EZH2.*

Data from murine models showed an interaction between ASXL1, RUNX1, EZH2 and BCL-2 expression [16–18]. Moreover, targeted genetic loss of *Asxl1* using a murine model (*Asxl1*^*-/-*^ Lin^-^ c-Kit^+^ cells) resulted in a significant down-regulation of *Bcl-2* [16]. These data suggested that BCL-2 inhibition might fail to kill progenitors in MDS that harbor any of these mutations. In contrary, recently published data showed an association of *ASXL1* mutations and elevated *HOX* gene expression in human *de novo* AML predicting sensitivity to BCL-2 inhibition [19]. This illustrates that the functional consequence of somatic mutations and the subsequent sensitivity to BCL-2 inhibition remain incompletely understood. The same holds true for mutations in *TP53*. The interplay between TP53 and BCL-2 has been appreciated for some time [20] and the impact of *TP53* mutations on the outcome of MDS patients is an extensively studied topic [21]. However, the impact of *TP53* mutations on members of the BCL-2 family and the response to BCL-2 inhibition in MDS has not been addressed.

Here we report on the impact of somatic mutations in *ASXL1*, *RUNX1*, *EZH2* or *TP53* on the sensitivity to ABT-199 treatment in patients with MDS and sAML. We find that ABT-199 effectively kills progenitor cells from high-risk MDS/sAML patients despite the presence of an adverse genetic profile suggesting that pro-apoptotic intervention will provide a therapeutic benefit to patients with high-risk MDS irrespective of their mutational status.

## RESULTS

### Gene expression of critical BCL-2 family members remain unaffected by mutations in *ASXL1*, *RUNX1*, *TP53* or *EZH2*

**Figure 1:**
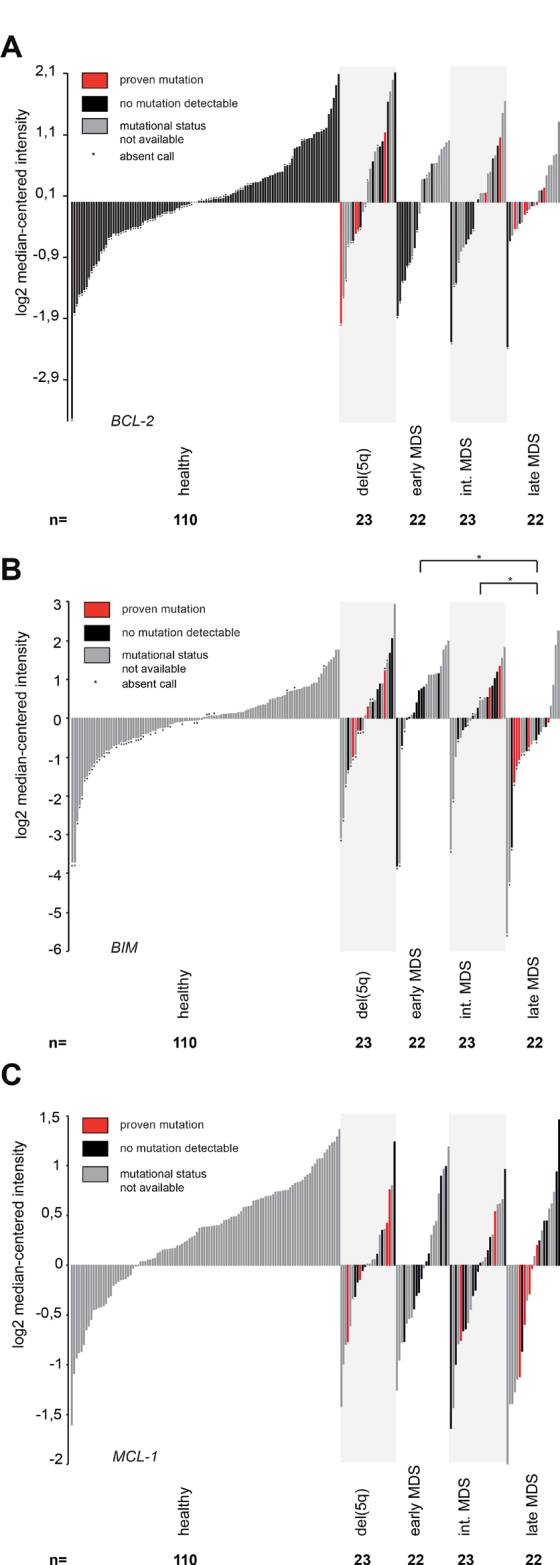
Gene expression analysis of critical BCL-2 family members shows reduced *BIM* expression in advanced MDS All panels: Gene expression was measured in 90 primary human MDS bone marrow samples and 110 healthy controls using the Human Genome U133 Plus 2.0 Array. Early MDS included MDS-SLD, MDS-MLD, MDS-RS-SLD and MDS-RS-MLD. MDS-EB-1 was defined as intermediate and MDS-EB-2 as late MDS. (**A**) Gene expression of *BCL-2* in primary human MDS samples and healthy controls (reporter 207004_at). Mutational status had no impact on BCL-2 expression in del (5q) (*p* = 0.16), MDS-EB-1 (*p* = 0.18) and MDS-EB-2 (*p* = 0.31). (**B**) Gene expression of *BIM* in primary human MDS samples and healthy controls (reporter 208536_s_at). Mutational status had no impact on BIM expression in del (5q) (*p* = 0.3), MDS-EB-1 (*p* = 0.26) and MDS-EB-2 (*p* = 0.82). *BIM* is downregulated in late MDS (MDS-EB-2) when compared to early MDS (*p* = 0.021) or intermediate MDS (*p* = 0.039), both irrespective of the mutational status. (**C**) Gene expression of *MCL-1* in primary human MDS samples and healthy controls (reporter 200798_x_at). Mutational status had no impact on MCL-1 expression in del (5q) (*p* = 0.81), MDS-EB-1 (*p* = 0.83) and MDS-EB-2 (*p* = 0.48).

To exclude an *a priori* resistance to ABT-199 by mutations in *ASXL1*, *RUNX1*, *TP53* or *EZH2*, we analysed gene expression data in an extended cohort of MDS patients (*n* = 90) with or without an adverse mutational profile and compared it to healthy controls (*n* = 110) (Figure 1). We were mainly interested in any gross aberrations of the gene expression levels of BCL-2 members that might preclude treatment response to ABT-199. Gene expression of *BCL-2* did not detect any differences between patients harbouring an adverse mutational profile to patients without this profile (Figure 1A). We also did not observe substantial differences in expression levels in MDS patients when compared to expression levels of healthy controls (Figure 1A). Moreover, we did not notice any relevant changes in expression in patients from lower-risk MDS to patients classified as higher-risk MDS (Figure 1A). Of note, functional analysis had shown a selective induction of apoptosis specifically in high-risk MDS patients [7]. We therefore concluded that the known sensitivity of high-risk MDS samples to ABT-199 [7] might not be determined by *BCL-2* mRNA expression alone, but might be caused by *BCL-2* protein levels or, alternatively, by expression of BCL-2 family members other than *BCL-2* itself.

We therefore analysed gene expression of BIM as a pro-apoptotic member of the BCL-2 family that effectively antagonizes BCL-2 and drives apoptosis induction. Previous reports had demonstrated that high-levels of BIM induce resistance to ABT-199 by prolonged stabilization of MCL-1 [22]. In our extended MDS cohort compared to earlier analyses [7] and in line with a skewed apoptotic balance in MDS [12, 13], our gene expression analysis of *BIM* showed a significantly decreased expression in advanced stages of MDS (MDS-EB-2) when compared to early MDS (MDS-SLD, MDS-MLD, MDS-RS-SLD, MDS-RS-MLD) (*p* = 0.021) or when compared to intermediate MDS (MDS-EB-1) (*p* = 0.039) (Figure 1B). The reduced expression of pro-apoptotic *BIM* in higher-risk MDS supported the notion that an acquired resistance to apoptosis occurred upon disease progression, which is in line with our cell culture-based data on ABT-199-induced apoptosis induction [7]. Of note, the *BIM* expression pattern was independent of the mutational profile suggesting that the presence or absence of individual somatic high-risk mutations in MDS might not alter *BIM* expression levels and therefore might not necessarily preclude the use of ABT-199 in these patients.

Next, we tested gene expression levels of *MCL-1* and *BCL-X*_*L*_ as the second most relevant pro-survival proteins from the BCL-2 family [23]. For *MCL-1*, previous publications had reported regulation of MCL-1 by TP53 [24] and EZH2 [25] and the interaction between MCL-1 and BIM had been suggested to predict sensitivity to BCL-2 inhibition [22, 26]. Hence, we analysed *MCL-1* gene expression levels but failed to identify any relevant differences in *MCL-1* gene expression between MDS subgroups. Moreover, we also did not find any difference in MCL-1 levels when comparing patients with or without an adverse mutational profile (Figure 1C). The same finding was observed for *BCL-X*_*L*_ (data not shown).

As gene expression levels only partially represent protein abundance in primary cells, we also performed protein quantification of BCL-2 in primary human BM stem/progenitor cells from MDS/sAML patients using flow cytometry to accurately reflect protein levels. In line with the gene expression data for *BCL-2* (Figure 1A), we found that the mutational status did not impact on BCL-2 protein levels in CD34^+^ stem/progenitor cells as measured by flow cytometry (Supplementary Figure 1). This implicated that BIM levels rather than BCL-2 levels induce the apoptotic resistance observed in higher-risk MDS.

In summary, the presence of adverse mutations including *ASXL1*, *RUNX1*, *EZH2* or *TP53* did not negatively impact on the gene expression profile of relevant BCL-2 family members suggesting that patients harboring a high-risk mutational profile might retain sensitivity to ABT-199.

### ABT-199 induces apoptosis in high-risk MDS and sAML despite the presence of an adverse mutational profile

Mutations in *ASXL1*, *RUNX1*, *TP53* or *EZH2* negatively impact on the prognosis of MDS patients and it remains unclear whether standard-of-care regimens are sufficient to induce long-lasting effects in this patient population. Therefore, we tested the apoptotic effect of ABT-199 in primary BM samples of 31 MDS/sAML patients presenting with mutations in *ASXL1*, *RUNX1*, *TP53* or *EZH2* in comparison to 21 non-mutated MDS samples (Table 1A and Supplementary Table 1). The MDS samples were compared to 10 age-matched BM samples obtained from hip replacement surgery from otherwise healthy individuals (Table 1B). In line with published literature [1], the negative impact of the mutational profile on life expectancy in our patient cohort was documented by the reduced overall survival rates of this patient cohort (Supplementary Figure 2). Regarding clinical parameters no relevant differences were detected between patients with or without an adverse mutational profile. Samples were collected from patients at first presentation or at any time point during treatment (Table 1C). For 29 patients, a full clinical follow-up was available (Supplementary Figure 2).

**Table 1:** Clinical characteristics of MDS patients contributing samples for *ex vivo* treatment with ABT-199

A							
IPSS category	sole *ASXL1* mutation	sole *RUNX1* mutation	sole *TP53* mutation	sole *EZH2* mutation	*ASXL1*/*RUNX1* mutation	*ASXL1*/*EZH2* mutation	*ASXL1*/*TP53* mutation
**MDS**	5	1	5	1	6	1	1
**low**	0	0	0	0	0	0	0
** int.-1**	5	1	1	1	4	1	1
** int.-2**	0	0	3	0	1	0	0
** high**	0	0	1	0	1	0	0
**sAML**	4	0	5	0	1	0	1

B						
IPSS category	no. of patients analyzed	mean age	female	male	unmutated	mutated
**total**	62	69.5	24	38		
**healthy**	10	58.1	6	4		
**MDS**	40	69.8	14	26	20	20
** low**	4	76.3	2	2	4	0
** int.-1**	22	65.8	7	15	8	14
** int.-2**	8	72.9	2	6	4	4
** high**	6	76.0	3	3	4	2
**sAML**	12	73.8	4	8	1	11

C							
therapy	MDS low	MDS int. I	MDS int. II	MDS high	sAML	unmutated	mutated
**therapy naive**	2	6	3	2	2	10	5
**under therapy**	2	12	5	2	7	7	21
** EPO**	2	4	0	0	0	3	3
**lenalidomide**	0	2	0	0	0	0	2
** HMA**	0	6	5	2	7	4	16
**HSCT (any time)**	0	2	1	2	0	2	3

(A) Distribution of mutations in *ASXL1*, *RUNX1*, *TP53* and *EZH2* in MDS and sAML samples. (B) Distribution of mean age, gender and mutational status of MDS/sAML patients and healthy controls. MDS risk groups were determined according to the IPSS. Patients with sAML were defined by ∼20% bone marrow blast infiltration. Mutational status of MDS and sAML samples was determined by conventional Sanger sequencing or next-generation sequencing. (C) Distribution of patients prior to treatment, patients who have already received any therapy prior to sample collection and patients with allogenous hematopoietic stem cell transplantation (HSCT) at any time during the course of the disease. Only patients with a complete follow-up were included in this analysis (*n* = 29). EPO = erythropoietin, HSCT = hematopoietic stem cell transplantation.

**Figure 2:**
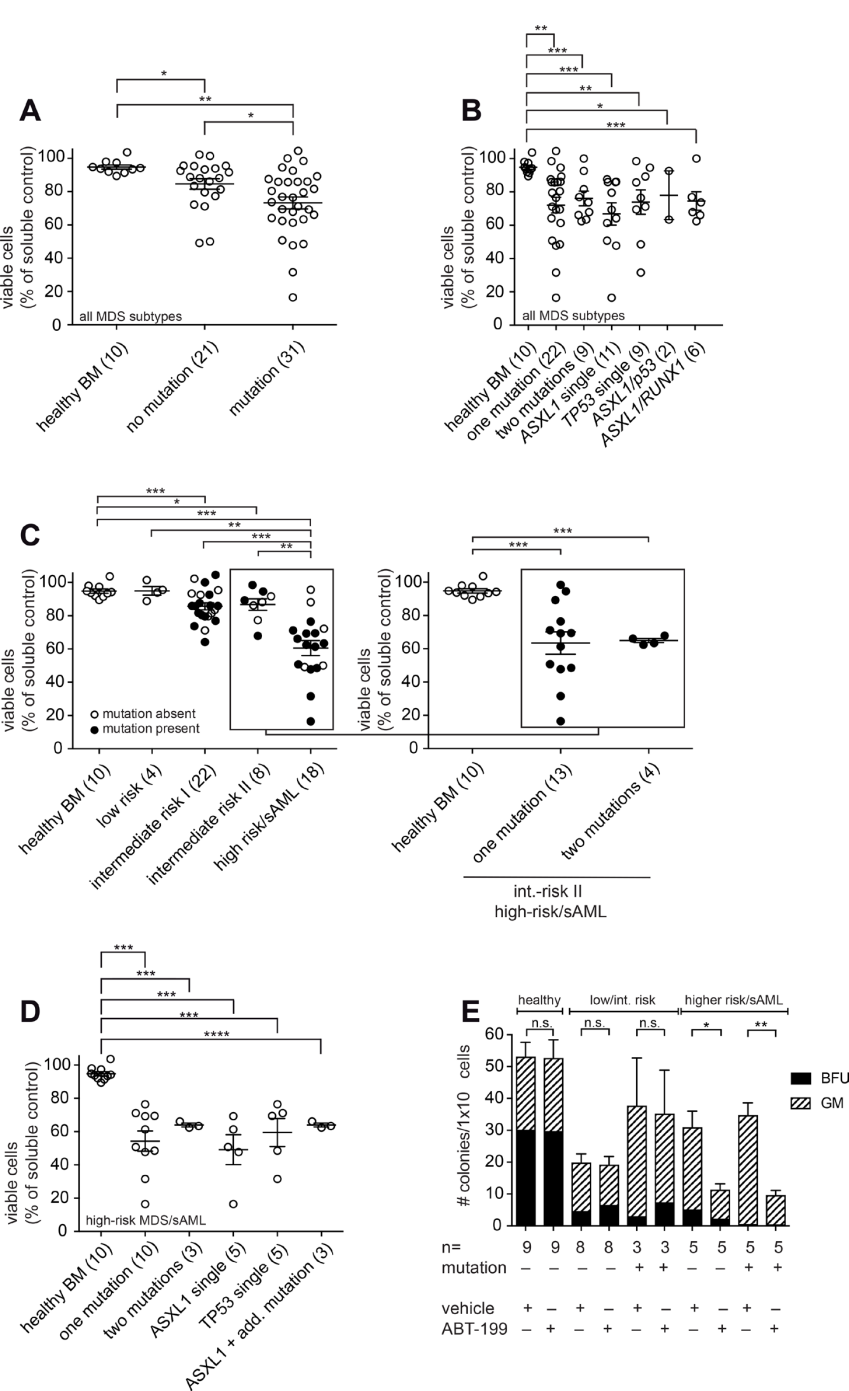
ABT-199 induces apoptosis in high-risk MDS stem/progenitor cells of patients with mutations in *ASXL1*, *RUNX1*, *TP53* or *EZH2* (**A**–**D**) BMMNC were treated with ABT-199 (1 μM) or vehicle control (DMSO) for 72 h. Viability of bone marrow stem/progenitor cells was measured by flow cytometry using Annexin V and 7AAD after gating on CD34^+^ cells. Each circle represents the ratio between viable cells (7AAD^−^ Annexin V^−^) after inhibitor treatment and viable cells after vehicle treatment. Shown is the mean and error bars denoting standard deviation. One-way ANOVA as indicated below and post-hoc pairwise *t*-tests as shown in the figure (^*^P < 0.05, ^**^P < 0.005 and ^***^P < 0.0005). Mean differences and 95% CI are listed in Supplementary Table 2. Patient samples were classified according to the IPSS. (A) BMMNC from healthy donors and MDS/sAML were treated with ABT-199 (1 μM) or vehicle control (DMSO) for 72 h. One-way ANOVA was *P* = 0.0011. (B) Viable BMMNC from 10 healthy donors and from MDS patients of all IPSS risk groups including 22 MDS/sAML patients with one mutation and 9 patients with two mutations in *ASXL1*, *RUNX1*, *TP53* or *EZH2* were treated with ABT-199 (1 μM) or vehicle control (DMSO) for 72 h. The most common mutations *ASXL1* single (*n* = 11), *TP53* single (*n* = 9), *ASXL1/TP53* (*n* = 2) and *ASXL1/RUNX1* (*n* = 6) are shown in detail. One-way ANOVA was *P* = 0.0224. (C) Viable BMMNC from healthy donors and MDS/sAML patients of the indicated risk groups were treated with ABT-199 (1 μM) or vehicle control (DMSO) for 72 h. One-way ANOVA was *P* < 0.0001. Then, the high-risk/sAML group was analysed in detail including 10 patients with one high-risk mutation and 3 patients with two high-risk mutations (including 2 patients with *ASXL1*+*RUNX1* mutation and 1 patient with *ASXL1*+*TP53* mutation). One-way ANOVA was *p* = 0.0007. (D) Viable BMMNC from 10 healthy donors, 10 high-risk MDS/sAML patients with one mutation and 3 high-risk/sAML patients with two mutations in *ASXL1*, *RUNX1*, *TP53* or *EZH2* were treated with ABT-199 (1 μM) or vehicle control (DMSO) for 72 h. The 3 high-risk/sAML patients with two mutations included 2 patients with *ASXL1*+*RUNX1* and 1 patient with *ASXL1*+*TP53*. One-way ANOVA was *P* = 0.0002. (**E**) BMMNC (1 × 10^4^) from healthy donors and MDS/sAML patients of the indicated risk groups and of the indicated mutational status were plated in methylcellulose after treatment with ABT-199 (1 μM) or DMSO for 72 h. Numbers of colony-forming units (CFU) of multi-potential granulocytic-erythroid-macrophagic-megakaryocytic lineage (CFU-GEMM), granulocytic-macrophagic lineage (CFU-GM), burst-forming units-erythroid (BFU-E) and total number of colonies were scored after 14 days. Experiments were performed in duplicates. Shown is the mean of the total colony numbers as stacked bar chart of the single colony types for the indicated risk groups. Error bars denote standard deviation. *P*-values as shown in the figure (^*^P < 0.05, ^**^ < 0.005).

We found that ABT-199 induced apoptosis significantly better in patients harbouring an adverse mutational profile (median viability 73.2%) when compared to non-mutated MDS patient samples (median viability 84.6%) and, alternatively, when compared to healthy controls (median viability 94.8%) (Figure 2A). By analysis of the different mutations in detail, we observed that ABT-199 induced apoptosis independently of the presence of one or two of the high-risk somatic mutations (Figure 2B). As shown in detail for the most common mutations (*ASXL1* single, *TP53* single, *ASXL1* and *TP53*, *ASXL1* and *RUNX1*) no individual mutation alone was capable of blocking ABT-199 efficacy (Figure 2B). It was curious to note that mutated samples underwent apoptosis better than non-mutated samples. This was unexpected as we had hypothesized that the presence of mutations might preclude ABT-199 effects. However, the difference likely originated from the elevated number of higher-risk MDS/sAML samples (by IPSS) in the mutational cohort (Table 1B). To exclude any bias in the analysis, samples from intermediate-risk and high-risk MDS/sAML samples bearing the same mutations were compared side-by-side. The direct comparison further illustrated that the effect of ABT-199 treatment was more pronounced in high-risk samples compared to intermediate-II risk samples and again remained unaffected by the mutational status (Supplementary Figure 3).

In summary, the presence of an adverse mutational profile does not restrict the treatment effect of ABT-199 in MDS progenitor cells.

### Efficacy of ABT-199 is observed in high-risk MDS/sAML irrespective of an adverse mutational profile

Patients clinically categorized as high-risk MDS or sAML have a poor prognosis [27] which is further impacted by an adverse mutational profile. It is therefore of particular interest to analyze the effect of ABT-199 in high-risk MDS patients that harbor adverse genetic mutations as these patients might benefit most from ABT-199.

To test the benefit of ABT-199 in this patient population, we directly compared induction of apoptosis in CD34^+^ stem/progenitor cells of patients with or without any of adverse mutations in correlation to the clinical risk score (Figure 2C). The induction of apoptosis was effective in mutated and in non-mutated samples specifically of the high-risk MDS/sAML cohort (Figure 2C). Even the presence of more than one mutation did not impair the apoptotic effect of ABT-199 in these patients (Figure 2C, insert on high-risk/sAML samples). Analysing the mutational profiles of high-risk MDS samples according to the presence of individual mutations, we found an equivalent efficacy in apoptosis induction by ABT-199 across the different genetic lesions (Figure 2D).

To understand the impact of the described mutations on the stem- and progenitor compartment after ABT-199 treatment, we analysed hematopoietic colony formation. Using high-risk MDS/sAML samples that carried one of the indicated adverse mutations, we observed a significant reduction in the total number of colonies generated from 1 × 10^4^ primary BM cells upon treatment with ABT-199 (Figure 2E). In line with our data from liquid culture, the presence of an adverse genetic profile did not preclude the killing effect of ABT-199 in longer-term colony assays supporting the notion that ABT-199 induces apoptosis in the stem- and progenitor compartment in MDS. Moreover, in patients presenting with an earlier disease stage of MDS, namely low- or intermediate-risk MDS with or without the high-risk mutations, we observed no significant reduction in colonies and no impact of the mutational status. We conclude that ABT-199 effectively targets the stem- and progenitor compartment in patients harbouring an adverse mutational profile.

### BCL-2 family members as potential biomarkers for ABT-199 treatment

**Figure 3:**
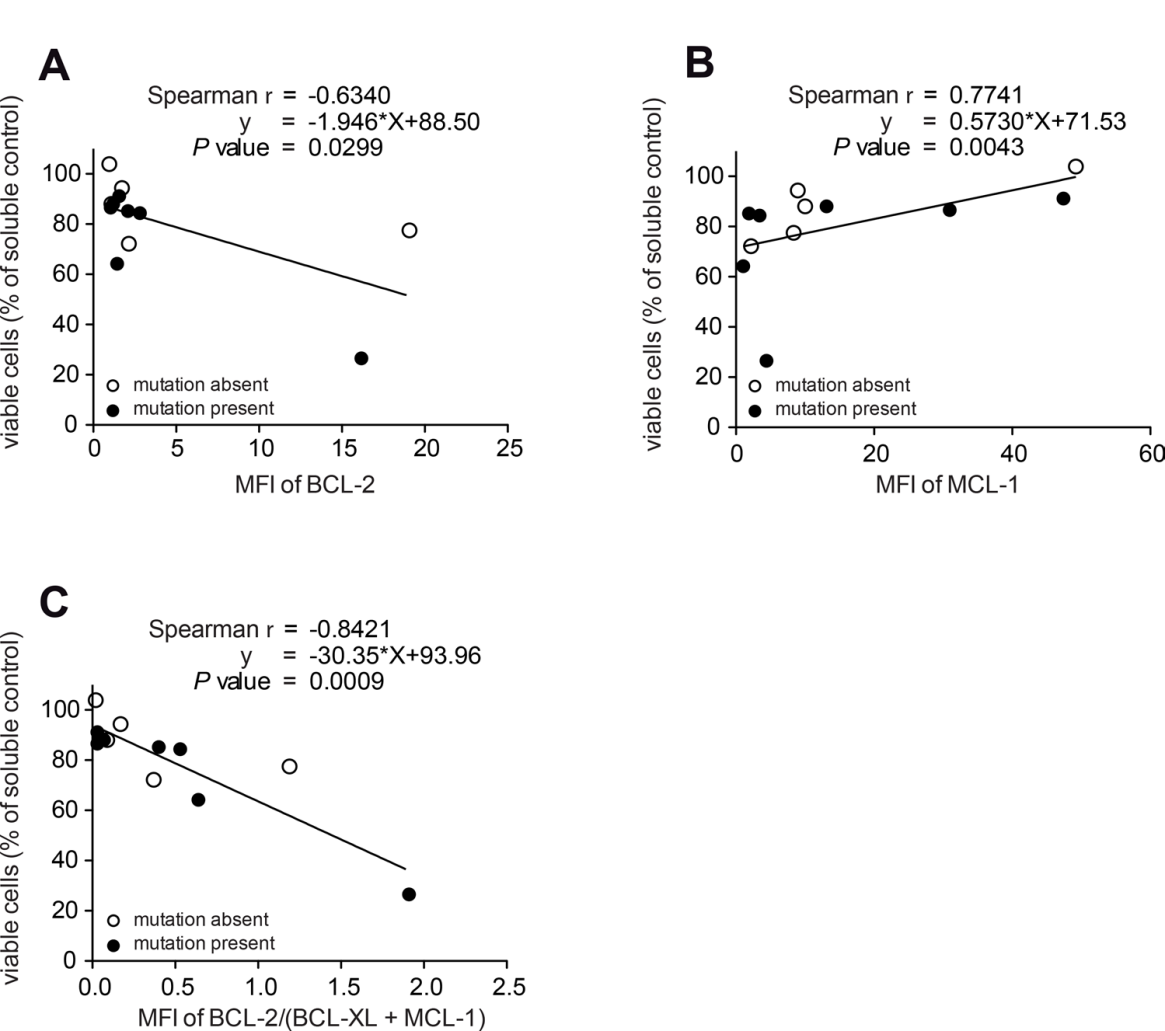
Detection of protein levels of BCL-2, BCL-XL and MCL-1 predict ABT- 199 response in patients with high-risk MDS All panels: BMMNC from 12 high-risk MDS/sAML patients with (*n* = 7) or without (*n* = 5) adverse mutational status were treated with ABT-199 (1 μM) or vehicle control (DMSO) for 72 h. Viability was measured as in Figure 2A. Each circle represents the ratio between viable cells after inhibitor treatment and viable cells after vehicle treatment for 72 h. Protein expression is shown by the mean fluorescence intensity (MFI) calculated as the ratio of stained antibody MFI devided by isotype control MFI by FACS. The strength of the association between MFI ratio and viability after inhibitor treatment was calculated by the Spearman rank correlation and the functional relationship was described by linear regression analysis. Measurement of BCL-2 (**A**) or MCL-1 (**B**) protein levels by flow cytometry. (**C**) Measurement of the ratio between BCL-2 and the combined value of BCL-XL and MCL-1 protein levels by flow cytometry in 12 high-risk MDS/sAML patients with (*n* = 7) or without (*n* = 5) adverse mutational status.

To evaluate potential biomarkers for the response of MDS samples with an adverse mutational profile to ABT-199, we correlated the abundance of critical BCL-2 proteins with cellular survival. The mean fluorescence intensity of the indicated proteins was measured by flow cytometry labelling intracellular proteins. The resulting MFI was then correlated with the viability after inhibitor treatment (Figure 3A). We specifically determined the protein expression in the subset of CD34^+^ stem/progenitor cells and focused on high-risk MDS/sAML patients. The strength of the association was calculated by the Spearman rank correlation and the functional relationship was described by linear regression analysis. This analysis showed that elevated levels of BCL-2 predicted good responses of ABT-199 (Figure 3A). Of note, high levels of MCL-1 were correlated with resistance to ABT-199 (Figure 3B) and the combination of the measurements of BCL-2, BCL-X_L_ and MCL-1 significantly improved the predictive power of this biomarker by flow cytometry (Figure 3C).

Together, our data showed that the protein levels of individual BCL-2 family members measured by flow cytometry can be used as biomarkers to define the differential sensitivity of MDS samples to the treatment with ABT-199 in high-risk MDS/sAML patients.

## DISCUSSION

Adverse somatic mutations including *ASXL1*, *RUNX1*, *TP53* or *EZH2* significantly impact on the prognosis of MDS patients [1] and established therapeutic concepts have only limited efficacy in this patient population [2–4]. It is therefore of critical importance to identify novel treatment options for these patients.

The potential impact of an adverse genetic profile on the use of ABT-199 is exemplified by preclinical data from a murine model that showed a significant downregulation of Bcl-2 in *Asxl1*^*-/-*^ progenitor cells [16]. Accordingly, loss of BCL-2 in patients harbouring this mutation might result in resistance to therapeutic BCL-2 inhibition. However the exact role of the putative tumor suppressor gene *ASXL1* in patients and its role in regulating apoptotic signalling pathways has not be fully delineated yet [28]. Hence, it is of considerable clinical relevance to understand whether the presence of adverse somatic mutations such as *ASXL1*, *RUNX1*, *TP53* or *EZH2* affect *BCL-2* expression and subsequently preclude the effect of ABT-199.

Our data showed that ABT-199 effectively induces apoptosis in high-risk progenitor cells despite the presence of adverse genetic aberrations. Especially the substantial reduction in the colony forming capacity after targeting the stem and progenitor compartment with ABT-199 emphasizes the clinical relevance of Venetoclax treatment in higher-risk MDS patients. Of note, even the presence of more than one adverse mutation did not preclude efficacy of ABT-199. This indicates that genetic mutations affecting epigenetic modifiers (such as *ASXL1* or *EZH2*), transcription factors (*RUNX1*) or *TP53* fail to overcome the dependency of the MDS progenitor cells on a survival signal mediated by BCL-2. Therapeutic intervention into the BCL-2 rheostat using ABT-199 tips the balance of these cells towards cell death despite their resistance to alternative anti-neoplastic treatment options such as hypomethylating agents.

In line with published incidence rates [29], our cohort included fewer patients with *EZH2* mutations than individuals with aberrations in *ASXL1*, *RUNX1* or *TP53* indicating that the effect of ABT-199 in samples with *EZH2* mutations might be under-represented. Combinations of *ASXL1* and *RUNX1* mutations were most commonly observed in the samples utilized for our study (Table 1A).

Despite the different functional consequences of the mutated genes, we did not detect any differences in the cytotoxic efficacy of ABT-199 between any of the individual aberrations. Furthermore, even the combination of several mutations did not negatively impact on the apoptotic efficacy of ABT-199. Hence, our data suggest that ABT-199 functions independently of specific signalling pathways by lowering the threshold for apoptotic induction in response to various cellular stresses.

This was especially interesting in samples with *TP53* or *RUNX1* mutations as both genes are involved in apoptotic signalling and deregulation was supposed to induce resistance to ABT-199 [30]. However, data obtained from patients with chronic lymphocytic leukemia support our finding by showing an excellent treatment effect of ABT-199 despite the presence of mutations in *TP53* [31].

The tight interplay between pro- and anti-apoptotic BCL-2 proteins regulates the threshold for mitochondrial apoptosis [11, 23, 32–36]. The presence of pro-apoptotic cues mediated by BH3-only proteins and the counterbalance by anti-apoptotic BCL-2 members together defines the apoptotic balance also termed “apoptotic priming”. In leukemic blasts, the level of “apoptotic priming” is relevant for treatment response and can be determined by BH3 profiling [37]. This includes the use of different BH3 peptides that elucidate the impact of individual members of the BCL-2 protein family on treatment resistance [38, 39]. “Apoptotic priming” illustrates that the susceptibility towards ABT-199 not only correlates with the amount of BCL-2 expression [7, 38], but is also influenced by the expression of additional members of the BCL-2 protein family that together define the apoptotic threshold. Accordingly, the understanding of potential treatment resistance to ABT-199 requires the analysis of pro- as well as anti-apoptotic BCL-2 members.

In line with this notion, our gene expression analysis showed high levels of pro-apoptotic *BIM* in lower-risk MDS patients. Hence, the apoptotic threshold in cells from these patients is relatively low and progression into more aggressive forms of the disease is, at least in part, prevented. Upon disease progression into higher-risk MDS, patients present with significantly reduced BIM expression and a subsequent resistance to apoptosis [9, 10, 12, 13, 15]. We conclude that the combined effects of pro- and anti-apoptotic BCL-2 members define the apoptotic susceptibility in primary human MDS. This is also exemplified by the finding that high expression levels of MCL-1 correlate with resistance to ABT-199 [7] and that MCL-1-specific siRNA increased the efficacy of ABT-199 in AML [40].

It remains unclear which individual biomarker best predicts treatment resistance to ABT-199. Our data showed that somatic mutations in *ASXL1, RUNX1, TP53* or *EZH2* did not preclude efficacy of ABT-199 in high-risk MDS. This indicates that patients with any of these mutations should not be precluded from treatment with ABT-199. Moreover, we showed a correlation between BCL-2 protein levels and the cytotoxic efficacy of ABT-199, which identified pre-treatment stratification by BCL-2 protein levels using flow cytometry as a feasible option for MDS patients. The combination of the MFI for BCL-2, MCL-1 and BCL-X_L_ strengthened the association calculated by the Spearman rank correlation further supporting the use of flow cytometry for this purpose. These data are in line with recently published findings in patients with T-cell prolymphocytic leukemia [41]. Here *ex vivo* responses to Venetoclax significantly correlated with BCL-2 protein expression scores. Expression of MCL-1 and BCL-X_L_ showed a clear tendency to negative correlation.

We conclude that selective BCL-2 inhibition is a promising target in high-risk MDS/sAML patients that harbour adverse mutations in *ASXL1*, *RUNX1*, *TP53* or *EZH2*.

## MATERIALS AND METHODS

### Patient samples

Human BM samples were collected according to the institutional guidelines and in concordance with the Declaration of Helsinki. Written informed consent was obtained from each patient. The investigation was approved by the Local Ethics Committee of the University Hospital of the Technical University in Munich. Samples were obtained when clinically required from patients either before or during treatment and irrespective of the therapeutic regimen. MDS patient samples were classified according to the International Prognostic Scoring System (IPSS) and sAML was defined as ≥ 20% of blasts in the BM and a history of MDS. Control samples for the use of ABT-199 *ex vivo* were obtained from human femoral heads discarded after implantation of total endoprosthesis of the hip joint from ten haematologically healthy age-matched donors.

### Cell isolation and culture 

Mononuclear cells from primary human BM samples were isolated via density-gradient centrifugation using the Biocoll Separation Solution (Biochrom AG, Berlin, Germany) following the manufacturer’s instructions. CD34^+^ cells were purified via positive selection using the CD34^+^ MicroBeads kit (Miltenyi Biotec, Bergisch Gladbach, Germany) and purity was confirmed to be at least 95%. BM mononuclear cells (BMMNC) were cultured at a density of 5 × 10^5^ cells/ml in serum-free media consisting of Iscove’s Modified Dulbecco’s Medium (IMDM) with L-alanyl-L-glutamine (IMDM GlutaMAX) with 20% BIT 9500 serum substitute (1% (w/v) bovine serum albumin, 10 μg/ml insulin, 200 μg/ml iron-saturated transferrin; StemCell Technologies, Vancouver, BC, Canada) and enriched with recombinant human stem cell factor (100 ng/ml), FMS-related tyrosine kinase-3 ligand (100 ng/ml), thrombopoetin (10 ng/ml), interleukin-6 (5 ng/ml), interleukin-3 (10 ng/ml; all from R&D Systems, Minneapolis, MN, USA), β-mercaptoethanol (10 μM; Gibco, Carlsbad, CA, USA) and low- density lipoproteins (4 μg/ml; Sigma-Aldrich, St Louis, MO, USA).

### Inhibitor 

ABT-199 (AbbVie, North Chicago, IL, USA) was dissolved in dimethyl sulfoxide (DMSO) and used in a final concentration of 1 μM. DMSO was used at 0.001% as vehicle control.

### Colony formation assay 

Hematopoietic progenitors were assessed after treatment with ABT-199 (1 μM) and DMSO (0.001%) for 72 h in cytokine-supplemented, serum-free culture. 1 × 10^4^ BMMNC were plated in duplicates in methylcellulose medium supplemented with an optimal cytokine mix according to the manufacturer’s protocols (MethoCult H4435 enriched; StemCell Technologies). Numbers of erythroid progenitor colonies (Burst-forming units-erythroid or colony-forming units for the granulocytic-macrophagic lineage, and multi-potential granulocytic-erythroid-macrophagic-megakaryocytic lineage) were assessed after 14 days. Transmitted light photographs were obtained on a Keyence BIOREVO BZ-900 microscope.

### Flow cytometry 

BMMNC were stained with AnnexinV-FITC in AnnexinV staining solution (0.1 M HEPES/NaOH, pH 7.4, 1.4 M NaCl 0.9%, 25 mM CaCl2), followed by staining with fluorescently labelled antibodies against CD34 (clone 4H11). For intracellular staining, cells were stained against CD34, followed by fixation in 2% paraformaldehyde, permeabilization using perm/wash buffer (BD Bioscience, Franklin Lakes, NJ, USA) and subsequent staining with fluorescently labeled antibodies against BCL-2 (clone Bcl-2/100, BD Bioscience) or respective isotype controls (Cat.: 556357, BD Bioscience; clone DA1E). Dead cells were excluded by Fixable Viability Dye staining. If not otherwise stated, reagents and antibodies were purchased from eBioscience. Flowanalysis was performed on a BD FACS Canto II (BD Bioscience) and data were analyzed using FlowJo software (TreeStar Inc., Ashland, OR, USA).

### Gene analysis

Mutational status of MDS and sAML samples was determined by conventional Sanger sequencing or next-generation sequencing at the MLL Munich Leukemia Laboratory. Patients were screened for mutations in *ASXL1*, *RUNX1*, *TP53* and *EZH2*.

### Gene expression analysis

Gene expression analysis was performed using the Human Genome U133 Plus 2.0 Array from Affymetrix (Santa Clara, CA, USA). The Affymetrix normalization method was used. All expression measurements of each array are divided by the median (calculated across all calls (present, mixed and absent)) and plotted on a logarithmic scale to normalize the data and show a log2 median-centered intensity blot. BMMNCs of 90 MDS patients (MDS with del (5q) *n* = 23; MDS-SLD *n* = 4, MDS-RS-SLD *n* = 6, MDS-MLD *n* = 6, MDS-RS-MLD *n* = 6, MDS-EB-1 (refractory anemia with blast excess 5–9%) *n* = 23, MDS-EB-2 (refractory anemia with blast excess ≥10%) *n* = 22) and peripheral blood mononuclear cells from 110 healthy controls were analyzed. To test for any significant differences of gene expression between the MDS groups, ‘early’, ‘intermediate’ and ‘late’ and in between the groups “proven mutation” vs “no mutation detectable” Student’s *T* Test was used. All reported *P*-values are two-sided, with a significance level of 0.05 and have not been adjusted for multiple testing (unpaired Student’s *t*-test). Pairwise differences are presented with 95% confidence intervals (CIs). Statistical analyses were performed using GraphPadPrism version 5.01 (Graphpad Software, Inc., San Diego, CA, USA).

### Statistical analysis 

To test for any significant differences in apoptosis induction after inhibitor treatment one-way ANOVA was used with post-hoc pairwise comparisons in case of significance. Pairwise differences are presented with 95% CIs. Comparing two samples, the unpaired Student’s *t*-test was used to test for any significant differences between treated samples and control. All reported *P*-values are two-sided, with a significance level of 0.05 and have not been adjusted for multiple testing. Statistical analyses were performed using GraphPadPrism version 5.01 (Graphpad Software, Inc., San Diego, CA, USA).

## SUPPLEMENTARY MATERIALS FIGURES AND TABLES


