# Supplementary material for: Selective inhibition of BCL-2 is a promising target in patients with high-risk myelodysplastic syndromes and adverse mutational profile
Source: Oncotarget. 2018 Apr 3;9(25):17270–81. doi: 10.18632/oncotarget.24775 (PMC5915115; doi:10.18632/oncotarget.24775)
Supplement: Supplementary file 1 [file oncotarget-09-17270-s001.pdf]

Selective inhibition of BCL-2 is a promising target in patients with high-risk myelodysplastic syndromes and adverse mutational profile

SUPPLEMENTARY MATERIALS

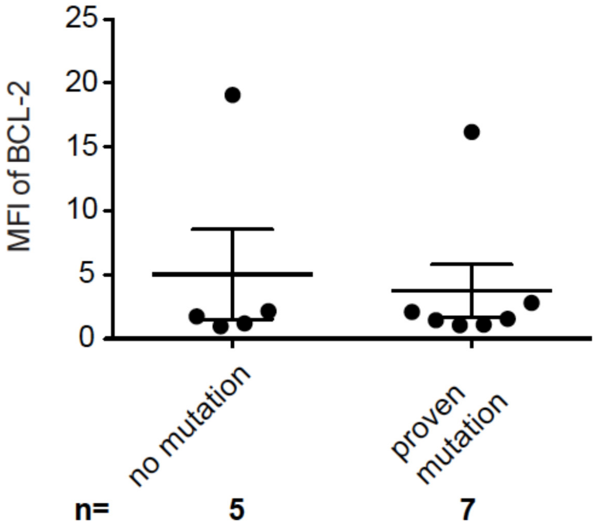

Supplementary Figure 1: Expression of BCL-2 in CD34⁺ MDS stem/progenitor cells is independent of the mutational status. This figure shows independency of BCL-2 protein expression from the mutational status in CD34⁺ MDS stem/progenitor cells measured by intracellular flow cytometry.

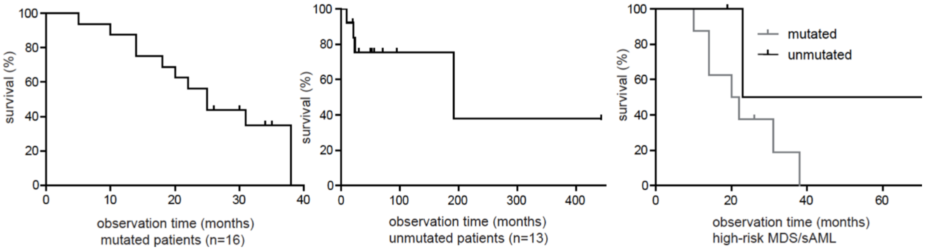

Supplementary Figure 2: Kaplan–Meier survival fractions of 29 patients with MDS/sAML whose BM samples were analyzed *ex vivo*. This graph shows survival of a number of patients treated with ABT-199 *ex vivo* providing sufficient clinical data.

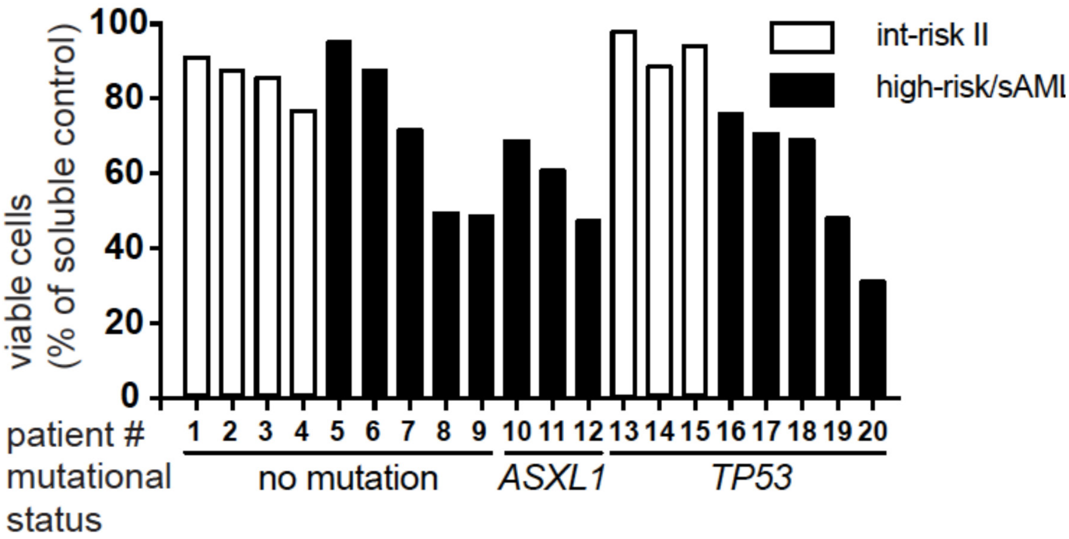

Supplementary Figure 3: ABT-199 selectively induces cell death in high-risk MDS/sAML progenitors irrespective of the mutational status. This figure shows head to head the viability of primary int.-risk II vs high-risk/sAML samples itemised by presence or absence of an adverse mutation.

Supplementary Table 1: Clinical characteristics of MDS and sAML patients contributing samples

Patient ID	Age	Gender	IPSS category	ASXL1 mutation	RUNX1 mutation	TP53 mutation	EZH2 mutation	Previous therapy
1	72	female	low	absent	absent	absent	absent	EPO
2	76	female	low	absent	absent	absent	absent	EPO
3	74	male	low	absent	absent	absent	absent	no therapy
4	83	male	low	absent	absent	absent	absent	no therapy
5	70	female	int.-1	absent	absent	absent	absent	no therapy
6	73	female	int.-1	absent	absent	absent	absent	no therapy
7	56	female	int.-1	absent	absent	absent	absent	n.a.
8	70	male	int.-1	absent	absent	absent	absent	no therapy
9	68	male	int.-1	absent	absent	absent	absent	n.a.
10	62	female	int.-1	absent	absent	absent	absent	no therapy
11	41	female	int.-1	absent	absent	absent	absent	EPO
12	67	male	int.-1	absent	absent	absent	absent	no therapy
13	87	male	int.-2	absent	absent	absent	absent	HMA
14	75	male	int.-2	absent	absent	absent	absent	no therapy
15	69	male	int.-2	absent	absent	absent	absent	HMA
16	57	male	int.-2	absent	absent	absent	absent	HMA
17	66	male	high	absent	absent	absent	absent	n.a.
18	86	female	high	absent	absent	absent	absent	n.a.
19	62	female	high	absent	absent	absent	absent	HMA
20	74	male	high	absent	absent	absent	absent	no therapy
21	76	male	sAML	absent	absent	absent	absent	no therapy
22	67	male	int.-1	present (n.d.)	absent	absent	absent	lenalidomide
23	74	male	int.-1	present (41%)	absent	absent	present (86%)	EPO
24	79	male	int.-1	present (40%)	present (30%)	absent	absent	EPO
25	64	female	int.-1	absent	absent	present (40%)	absent	lenalidomide
26	74	male	int.-1	present (6%)	present (16%)	absent	absent	HMA
27	63	male	int.-1	present (n.d.)	absent	absent	absent	no therapy
28	61	female	int.-1	absent	present (n.d.)	absent	absent	HMA
29	66	male	int.-1	present (n.d.)	absent	absent	absent	n.a.
30	80	male	int.-1	present (40%)	absent	absent	absent	HMA
31	66	male	int.-1	present (n.d.)	absent	absent	absent	EPO
32	75	male	int.-1	present (n.d.)	present (n.d.)	absent	absent	HMA
33	60	male	int.-1	absent	absent	absent	present (n.d.)	n.a.
34	43	male	int.-1	present (n.d.)	absent	present (n.d.)	absent	HMA
35	68	male	int.-1	present (n.d.)	present (n.d.)	absent	absent	HMA
36	73	female	int.-2	absent	absent	present (n.d.)	absent	no therapy
37	69	male	int.-2	present (50%)	present (50%)	absent	absent	no therapy
38	73	female	int.-2	absent	absent	present (n.d.)	absent	HMA
39	80	male	int.-2	absent	absent	present (n.d.)	absent	HMA
40	94	female	high	absent	absent	present (40%)	absent	no therapy
41	74	male	high	present (50%)	present (50%)	absent	absent	HMA
42	78	male	sAML	present (50%)	absent	present (63%)	absent	HMA
43	75	male	sAML	absent	absent	present (53%)	absent	no therapy
44	81	male	sAML	present (6%)	absent	absent	absent	HMA
45	82	male	sAML	present (6%)	absent	absent	absent	HMA
46	65	female	sAML	absent	absent	present (61%)	absent	HMA
47	85	female	sAML	absent	absent	present (53%)	absent	HMA
48	71	male	sAML	present (11%)	absent	absent	absent	n.a.
49	79	female	sAML	absent	absent	present (49%)	absent	n.a.
50	74	female	sAML	absent	absent	present (n.d.)	absent	HMA
51	68	male	sAML	present (n.d.)	present (n.d.)	absent	absent	HMA
52	52	male	sAML	present (n.d.)	absent	absent	absent	n.a.

Age, gender, IPSS category and the presence or absence of mutations in ASXL1, RUNX1, TP53 or EZH2 were documented for each patient sample Patients with sAML were defined by ≥ 20% bone marrow blast infiltration.
Mutational status of MDS and sAML samples was determined by conventional Sanger sequencing or next-generation sequencing. If available, mutation load is indicated as percentage.

Supplementary Table 2: Differences of mean with 95% CI. In addition to the statistical analysis shown in Figure 2 mean differences are presented with 95% confidence intervals

Figure	Bonferroni's Multiple Comparison Test	Mean differences	95% CI
Figure 2A	mutation (31) vs. no mutation (21)	–11.41	–22.88 to 0.0703
	mutation (31) vs. healthy BM (10)	–21.58	–36.35 to –6.81
	no mutation (21) vs. healthy BM (10)	–10.17	–25.77 to 5.431
Figure 2B	healthy BM (10) vs. one mutation (22)	22.75	–1.756 to 47.25
	healthy BM (10) vs. two mutations (9)	18.72	–10.79 to 48.24
	healthy BM (10) vs. ASXL1 single (11)	27.99	–0.07816 to 56.06
	healthy BM (10) vs. TP53 single (9)	20.88	–8.642 to 50.39
	healthy BM (10) vs. ASXL1/TP53 single (2)	16.83	–32.93 to 66.59
	healthy BM (10) vs. ASXL1/RUNX1 (6)	20.29	–12.89 to 53.46
	one mutation (22) vs. two mutations (9)	–4.02	–29.44 to 21.4
	one mutation (22) vs. ASXL1 single (11)	5.246	–18.48 to 28.97
	one mutation (22) vs. TP53 single (9)	–1.869	–27.29 to 23.55
	one mutation (22) vs. ASXL1/TP53 single (2)	–5.913	–53.36 to 41.53
	one mutation (22) vs. ASXL1/RUNX1 (6)	–2.457	–32.04 to 27.13
	two mutations (9) vs. ASXL1 single (11)	9.267	–19.61 to 38.14
	two mutations (9) vs. TP53 single (9)	2.151	–28.13 to 32.44
	two mutations (9) vs. ASXL1/TP53 single (2)	–1.893	–52.11 to 48.33
	two mutations (9) vs. ASXL1/RUNX1 (6)	1.564	–28.63 to 31.76
	ASXL1 single (11) vs. TP53 single (9)	–7.116	–35.99 to 21.76
	ASXL1 single (11) vs. ASXL1/TP53 single (2)	–11.16	–60.54 to 38.22
	ASXL1 single (11) vs. ASXL1/RUNX1 (6)	–7.703	–36.78 to 21.37
	TP53 single (9) vs. ASXL1/TP53 (2)	–4.044	–54.26 to 46.18
	TP53 single (9) vs. ASXL1/RUNX1 (6)	–0.5872	–34.45 to 33.27
	ASXL1/TP53 single (2) vs. ASXL1/RUNX1 (6)	–3.457	–55.91 to 49.00
Figure 2C	healthy BM (10) vs. low-risk (4)	–0.148	–22.03 to 21.73
	healthy BM (10) vs. intermediate-risk I (22)	9.165	–4.939 to 23.27
	healthy BM (10) vs. intermediate-risk II (8)	8.125	–9.417 to 25.67
	healthy BM (10) vs. high-risk/sAML (18)	34.25	19.66 to 48.83
	low-risk (4) vs. intermediate-risk I (22)	9.313	–10.79 to 29.41
	low-risk (4) vs. intermediate-risk II (8)	8.272	–14.37 to 30.92
	low-risk (4) vs. high-risk/sAML (18)	34.4	13.95 to 54.84
	intermediate-risk I (22) vs. intermediate-risk II (8)	–1.041	–16.31 to 14.23
	intermediate-risk I (22) vs. high-risk/sAML (18)	25.08	13.33 to 36.84
	intermediate-risk II (8) vs. high-risk/sAML (18)	26.12	10.41 to 41.84
Figure 2C	healthy BM (10) vs. one mutation (13)	31.32	12.6 to 50.04
(small)	healthy BM (10) vs. two mutations (4)	29.85	3.517 to 56.18
	one mutation (13) vs. two mutations (4)	–1.473	–26.92 to 23.98
Figure 2D	healthy BM (10) vs. one mutation (10)	40.5	19.47 to 61.54
	healthy BM (10) vs. two mutations (3)	30.83	–0.1301 to 61.79
	healthy BM (10) vs. ASXL1 single (5)	45.67	19.91 to 71.43
	healthy BM (10) vs. TP53 single (5)	35.34	9.574 to 61.1
	healthy BM (10) vs. ASXL1 + add. mutation (3)	30.83	–0.1301 to 61.79
	one mutation (10) vs. two mutations (3)	–9.674	–40.63 to 21.29
	one mutation (10) vs. ASXL1 single (5)	5.169	–20.59 to 30.93
	one mutation (10) vs. TP53 single (5)	–5.169	–30.93 to 20.59
	one mutation (10) vs. ASXL1 + add. mutation (3)	–9.674	–40.63 to 21.29
	two mutations (3) vs. ASXL1 single (5)	14.84	–19.5 to 49.19
	two mutations (3) vs. TP53 single (5)	4.505	–29.84 to 38.85
	two mutations (3) vs. ASXL1 + add. mutation (3)	0.00	–38.4 to 38.4
	ASXL1 single (5) vs. TP53 single (5)	–10.34	–40.08 to 19.41
	ASXL1 single (5) vs. ASXL1 + add. mutation (3)	–14.84	–49.19 to 19.5
	TP53 single (5) vs. ASXL1 + add. mutation (3)	–4.505	–38.85 to 29.84
